# COVID-19-Related Pathologies in Coronary Angiography in Patients with Acute Coronary Syndromes

**DOI:** 10.3390/jcm14113672

**Published:** 2025-05-23

**Authors:** Karolina Skonieczna, Olimpia Wiciun, Katarzyna Pinkowska, Tomasz Dominiak, Klaudyna Grzelakowska, Michał Kasprzak, Paweł Szymański, Jacek Kubica, Piotr Niezgoda

**Affiliations:** 1 Student Research Club of Cardiology, L. Rydygier Collegium Medicum in Bydgoszcz, Nicolaus Copernicus University in Torun, 85-027 Bydgoszcz, Polandpinkowska90@gmail.com (K.P.); 2 Department of Cardiology and Internal Medicine, L. Rydygier Collegium Medicum in Bydgoszcz, Nicolaus Copernicus University in Torun, 85-094 Bydgoszcz, Poland; lek.tomasz.dominiak@gmail.com (T.D.); klaudyna.grzelakowska@gmail.com (K.G.); medkas@o2.pl (M.K.); jkubica@cm.umk.pl (J.K.); piotr.niezgoda1986@gmail.com (P.N.); 3 Department of Cardiology, Invasive Cardiology and Electrophysiology with Intensive Cardiac Care Subunit, Regional Specialist Hospital, 86-300 Grudziadz, Poland; pawel.szymanski9@gmail.com

**Keywords:** COVID-19, ACS, coronary angiography, PCI, hypercoagulability

## Abstract

**Background:** The SARS-CoV-2 virus, identified in December 2019, led to a global pandemic resulting in over 6 million deaths. While most COVID-19 cases present mild symptoms, severe complications can develop in immunocompromised patients, including impacts on the heart. This study aimed to compare angiographic findings and hospitalization outcomes in acute coronary syndrome (ACS) patients with and without COVID-19. **Methods:** This retrospective study analyzed 174 ACS patients (105 men, 69 women) hospitalized in the Department of Cardiology and Internal Medicine of the Nicolaus Copernicus University in Bydgoszcz and Regional Hospital in Grudziądz (2019–2021). Forty-eight of them had COVID-19. The analyzed parameters included, inter alia, the coronary artery disease severity, the presence of thrombosis, survival rates, risk factors, and prior endovascular procedures. **Results:** COVID-19 patients with ACS showed a higher rate of thrombus in non-culprit vessels (6.25% vs. 0.0%, *p* = 0.0293), and overall survival was significantly lower (68.75% vs. 93.65%, *p* < 0.0001), while prior PCI rates were higher in non-COVID patients (34.13% vs. 6.25%, *p* = 0.0002). Procedure times were shorter for non-COVID patients, reducing catheterization lab exposure. Other procedural factors showed no significant differences. **Conclusions:** This study highlights significant differences in coronary angiography and hospitalization outcomes between ACS patients with and without COVID-19. The extended stay of COVID-19 patients in the catheterization lab poses an increased risk to medical staff, and the presence of thrombi underscores the need for effective antithrombotic strategies. The significant association of COVID-19 with hypercoagulability and its role in precipitating acute coronary syndromes necessitates the development of specific clinical guidelines to manage these patients effectively.

## 1. Introduction

SARS-CoV-2, discovered in December 2019, led to a global pandemic of the coronavirus disease 2019 (COVID-19), causing over 6 million deaths worldwide [1]. Most COVID-19-positive patients present mild respiratory symptoms such as fever, fatigue, headache, sore throat, cough, anosmia and gastrointestinal issues. However, studies have shown that patients with immunodeficiency and other comorbidities may progress to more severe illness, developing symptoms in other organs such as the liver, kidneys, heart and central nervous system [2].

One of the manifestations of severe COVID-19 is acute coronary syndrome (ACS) [3], the presentations of which comprise ST elevation myocardial infarction (STEMI), non-ST elevation myocardial infarction (NSTEMI) and unstable angina. In the majority of cases, it is caused by a plaque rupture in one of the coronary arteries [4]. Studies conducted during the COVID-19 pandemic have shown cases of ACS with normal coronary arteries via angiography [5], as well as with thromboembolism [6]. The mechanisms of ACS development during severe SARS-CoV-2 infection are not yet confirmed; however, they are thought to be related to high levels of proinflammatory cytokines, oxidative stress and endothelial dysfunction [7,8], resulting in hypercoagulability and leading to the development of intracoronary microthrombi or thrombus formation (type 1 myocardial infraction). It is thought that unobstructed coronary vessels in some COVID-19-positive patients with ACS may be a result of an oxygen supply–demand imbalance caused by respiratory failure (type 2 myocardial infarction) [9].

Coronary angiography allows us to detect some types of abnormalities in coronary arteries; as such, examining (or evaluating) the angiographic images of patients with COVID-19 could contribute to a better understanding of the mechanisms leading to the development of ACS in COVID-19, and further influence its treatment and prevention.

Due to the limited data on the use of coronary angiography in COVID-19-positive patients with ACS, we conducted a study aiming to determine specific features in coronary angiography in patients with ACS and coexisting COVID-19, and potential significant differences in the clinical outcomes of therapeutic procedures when compared to patients with ACS before the COVID-19 pandemic.

## 2. Methods

This is a retrospective cross-sectional study in which patients with a clinical diagnosis of ACS and COVID-19 infection were compared to ACS patients without COVID-19 infection in terms of coronary angiography upon admission. The study and control groups consisted of patients hospitalized in the Department of Cardiology and Internal Medicine of the Nicolaus Copernicus University, Bydgoszcz, Poland and in Department of Cardiology, Invasive Cardiology and Electrophysiology with Intensive Cardiac Care Subunit, Regional Specialist Hospital, Grudziądz, Poland from 1 January 2019 to 31 December 2021. COVID-19-positive patients enrolled in the study were hospitalized between October 2020 and December 2021, whereas COVID-19-negative patients were enrolled from April to May of 2019. All the data were obtained from electronic medical records. Several characteristics related to the severity of the coronary artery disease and treatment methods applied, with a focus on the outcome of the procedure, were assessed in order to compare the groups. The severity of coronary artery involvement was determined by analysis of the computer records derived from coronary angiography and based on the total number of coronary vessels with significant obstruction. Accordingly, patients were divided into four groups: those with left main coronary artery involvement alone, and those with the occlusion of one, two or three coronary arteries, with an additional criterion being the presence of coronary thrombosis within the vessels assessed by at least two authors. Additionally, an analysis of the clinical outcome of the treatments based on mortality rates during the hospitalization was performed. Further follow-up of the survivability rates was not conducted. Due to the retrospective character of the analysis, no informed consent was required from participants.

The statistical analysis was carried out using the Statistica v.13.0 package (TIBCO Software Inc., San Ramon, CA, USA) and PQStat v.1.8 (PQStat Software, Poznan, Poland). Continuous variables were presented as means with standard deviations. The Shapiro–Wilk test demonstrated non-normal distribution of the continuous variables under investigation. Therefore, non-parametric tests were used for statistical analysis. Comparisons between groups were performed with the Mann–Whitney unpaired rank sum test. Categorical variables were expressed as the number and the percentage. Categorical variables were compared using the χ^2^ test, χ^2^ test with Yeats’ correction or Fisher’s exact test, depending on the group size. Due to multiple comparisons, the Benjamini–Hochberg correction was applied. Results were considered significant at *p* < 0.05. To identify the predictor variables for mortality univariate and multivariate logistic regression models were used. Variables with a *p* value < 0.1 in the univariate analysis were introduced into the multivariate logistic regression model. In order to select the best model, the stepwise backward regression method was applied. Subsequently, variables without significant impact (*p* ≥ 0.05) were successively removed from the multivariate model according to their decreasing *p* values.

## 3. Results

A total of 174 participants (105 males [63.79%] and 69 females [36.21%]) were included in the analysis. Of them, 48 patients (27.6%) presented with COVID at admission. The median ages of the participants were 70 years (62.0–79.0) and 76 years (67.0–83.0) for non-COVID and COVID-positive patients, respectively (*p* = 0.0322). The rate of prior PCI was significantly higher in non-COVID patients than in the COVID group (43 patients [34.13%] vs. three patients [6.25%], *p* = 0.0002). Apart from the differences in age and rate of prior PCI, the study population was well-balanced. The baseline population’s characteristics are presented in Table 1.

Regarding the procedure, the preparation time, measured between entering the cathlab and the beginning of the procedure, was shorter for non-COVID patients (10.0 min [10.0–10.0] vs. 15.0 min [10.0–22.5], *p* < 0.0001). Moreover, the procedure itself was significantly shorter in this group (25.0 min [15.0–40.0] vs. 35.0 min [15.0–47.5], *p* = 0.0468). Significant differences were also observed in the time between the end of the procedure and the moment of departure from the cathlab (5.0 min [5.0–5.0] vs. 15 min [10.0–25.0], *p* < 0.0001 for non-COVID and COVID-positive patients, respectively). The overall time between entering and leaving the cathlab was also significantly shorter in the non-COVID population than in the COVID group (40 min [30.0–60.0] vs. 65 min [57.5–100.0], *p* < 0.0001). The doses of radiation and contrast medium used during the procedure did not differ significantly. A summary of the procedural parameters is presented in Table 2.

The evaluation of angiographic results has not revealed a significant difference in the rates of thrombus in anything other than a culprit vessel (6.25% vs. 0.0%, *p* = 0.1905) and the thrombus on an atherosclerotic plaque in COVID-positive patients compared to non-COVID patients (10.42% vs. 7.14%, *p* = 0.9608). Blood flow in the culprit vessel, assessed at the TIMI scale, did not differ significantly between the study groups either before or after the angioplasty. Nevertheless, a trend toward lower grades was observed in COVID patients (*p* = 0.3610). The overall survival rate was significantly lower in the COVID arm (68.75% vs. 93.65%, *p* < 0.0001). All the remaining data obtained during coronary angiography, including the rate of left main trunk disease, one-, two- and three-vessel disease, thrombectomy use, glycoprotein IIb/IIIa (GpIIb/IIIa) use, the no-reflow phenomenon and dissection after PCI, show no significant differences (Table 3).

Univariate and multivariate logistic regression for predictors of mortality is presented in Table 4.

## 4. Discussion

To the best of our knowledge, the study discussed herein is the largest to compare differences in hospitalization and angiographic data between patients with acute coronary syndrome suffering from COVID-19 and those who were COVID-19-negative. The temporal mismatch between the control and study groups was intentional to avoid inadvertent failure to detect SARS-CoV-2 infection, however it may introduce some inaccuracies.

Particular attention should be paid to the significant differences, revealed for the first time in our study, regarding the longer stay of COVID-positive patients in the catheterization laboratory. Each of the time factors analyzed, including the time required for the preparation of the patient and the time of the procedure itself, were extended. Moreover, the moment of departure from the cathlab was delayed, resulting in a prolonged total stay of patients with COVID-19 in the laboratory and, consequently, increased exposure of medical staff to the disease. This extended duration of procedural phases in COVID-19-positive patients is notable, as time delays in revascularization are recognized predictors of mortality. While in our multivariate analysis, these factors did not emerge as independent predictors, age and COVID-19 status were both significant. Since older patients would not necessarily require longer cathlab stays, this finding suggests that the additional delays were directly attributed to the presence of COVID-19 infection. These procedural inefficiencies may have played a role in the unfavorable outcomes observed in ACS patients with COVID-19. Interestingly, a longer percutaneous coronary intervention in patients with SARS-CoV-2 infection was not associated with an increase in the amount of contrast agent and radiation used in the procedure compared to patients without this infection. The prolonged exposure of medical staff to a COVID-19-positive patient is positively correlated with the results presented by Grzelakowska [10], which indicate an increased incidence of infections and quarantines in medical workers compared to the general population. The absence of employees due to illness or isolation, manifesting a reduction in hospital resources, was one of the factors contributing to the limited admissions of patients to hospitals during the pandemic [11]. Overall, in Poland, hospitalizations in cardiology departments decreased by 28.6% in 2020, compared to the previous year [12]. These changes to and postponements of elective procedures related to cardiovascular diseases have directly resulted in an increase in the total number of out-of-hospital deaths due to cardiovascular diseases [13].

COVID-19 has a close correlation with a state of general hypercoagulability. However, the relationship between this disease and the occurrence of blood clots is not entirely well defined. It is believed that this phenomenon may be influenced by endothelial dysfunction, the activation of cytokines and inflammatory mediators, or blood stasis leading to an imbalance between pro- and antithrombotic factors, which in turn predisposes the patient to the occurrence of disseminated intravascular coagulation (DIC), arterial thrombosis or venous thromboembolism [6]. In our study, a significant difference in the use of thrombectomy or GpIIb/IIIa inhibitors was not observed, nor was an increased amount of thrombi in coronary arteries in patients with COVID-19. However, such results can be found in other studies [6,14,15,16]. These results imply that the presence of COVID-19 does not necessarily confer more severe thrombotic tendencies or a greater thrombus burden than in non-COVID patients. Instead, the poorer outcomes observed in COVID-19-positive ACS patients may be explained by the procedural delays in preparation and revascularization, which may have heightened the risk of mortality. The large number of thromboembolic complications arising in the course of SARS-CoV-2 infection may impact the development of effective strategies incorporating the use of outpatient antithrombotic prophylaxis in infected patients, as well as impacting assessments of its safety [17]. Ning Tang’s study proves that the application of such a therapy in patients with severe COVID-19, and meeting the criteria of sepsis-induced coagulopathy or possessing an elevated D-dimer concentration, is associated with a better prognosis [15]. Particular care should be given to patients admitted to the intensive care unit who have the highest risk of developing this type of complication [18]. Additionally, recent studies show that SARS-CoV-2 infection is associated with possible persistent side effects on the vascular system, such as chronic endothelial dysfunction and microthrombosis, which may lead to cardiovascular manifestations [19].

During the pandemic, an increased incidence of acute coronary syndromes was observed, and this is directly linked with COVID-19 infection [20]. It was noticed that the occurrence of a generalized cytokine storm influenced the faster formation of atherosclerotic plaques and the destabilization of existing ones, as well as an increased coronary flow, which led to raised shear stress [6,21]. The occurrence of these mechanisms of ACS in the course of COVID-19 explains why, in our study, we did not find statistically significant differences between the groups of patients in terms of the incidence of thrombi within atherosclerotic plaques in a vessel undergoing PCI. We also did not identify a relationship between the occurrence of the disease and the number of vessels affected by atherosclerotic plaques. Another study noted that as many as 1/3 of patients with COVID-19 and ACS have normal arteries according to angiography, which may indicate the silent rupturing or erosion of atherosclerotic plaque, along with microthrombi or hypoxia [5]. Our study shows that patients without viral infection while suffering from ACS were more likely to have undergone PCI in the past, which means that their cardiovascular disease had already been revealed. It can be assumed that COVID-19 is thus a primary causative factor in the rupturing of pre-existing atherosclerotic plaques and the development of symptoms of ACS in patients who would possibly not have experienced them otherwise.

During the pandemic, some researchers began to recommend a more conservative approach to the treatment of ST elevation ACS using fibrinolysis, due to the proven susceptibility to hypercoagulability in COVID-19. However, it has been proven that despite the more frequent occurrence of clots in coronary vessels in patients with COVID-19, fibrinolysis is less effective than primary PCI. Therefore, as long as an invasive treatment with the option for revascularization can be safely performed, this approach should be recommended [22]. PCI reduces the risk of re-infarction and improves patient prognosis [22,23]. This is extremely important due to the statistically significantly higher death rate of patients suffering from COVID-19.

## 5. Conclusions

In this study, we compared angiographic findings and data on the overall time spent in the catheterization lab between COVID-19-positive and non-COVID-19 patients with acute coronary syndrome. Patients infected with SARS-CoV-2 underwent an extended stay in the catheterization lab, which increased the exposure of medical staff to the disease. Although our study did not show a higher rate of thrombosis in the vessels in COVID-19 patients with ACS, a link between the emergence of hypercoagulability during SARS-CoV-2 infection and its course of ACS with a higher mortality rate should be investigated. Moreover, while statistical significance for preparation time, procedure duration, and cathlab stay disappeared in the multivariate model with COVID-19 infection as a covariate, the observed delays in revascularization and post-PCI care suggest that SARS-CoV-2 infection may have contributed to worse clinical outcomes through indirect mechanisms. The extended procedural time could be a reflection of additional logistical challenges posed by COVID-19, including increased precautionary measures, staff shortages, and the necessity for enhanced patient management protocols. These delays may have exacerbated ischemic burden, thus increasing the risk of mortality in ACS patients. This interplay between delayed treatment and worse prognosis in ACS patients with COVID-19 warrants further investigation. Further research is needed to better understand the cardiovascular outcomes of COVID-19 so as to implement safe antithrombotic prevention strategies and the effective treatment of resulting changes.

## Figures and Tables

**Table 1:** Baseline characteristics of the study population.

	Non-COVID *	COVID (+) *	*p*-Value
Age	70.0 (62.0–79.0)	76.0 (67.0–83.0)	0.1610
Female	50 (39.7)	19 (39.6)	0.9905
Weight	80.0 (70.0–88.0)	82.0 (72.0–98.0)	0.2905
Height	170.0 (163.0–175.0)	170.0 (164.0–174.0)	0.9678
BMI	27.7 (24.8–30.8)	29.04 (25.5–34.0)	0.2905
History of diabetes mellitus	44 (34.9)	19 (39.6)	0.8104
History of arterial hypertension	90 (71.4)	34 (70.8)	0.9905
Prior myocardial infarction	42 (33.3)	13 (27.1)	0.7135
Prior PCI	43 (34.1)	3 (6.3)	0.0020
Prior CABG	7 (5.6)	0 (0.0)	0.4334

* The data are presented as the median (interquartile range).

**Table 2:** Procedural parameters between the study participants.

	Non-COVID (N = 126) *	COVID (+) (N = 48) *	*p*-Value
Preparation time [min]	9.7 ± 2.7	19.3 ± 11.2	<0.0001
Procedure time [min]	29.6 ± 19.9	40.3 ± 28.7	0.0468
Leaving time (end of procedure to departure) [min]	6.1 ± 2.6	22.3 ± 23.9	<0.0001
Overall cathlab time [min]	45.4 ± 20.8	83.1 ± 47.4	<0.0001
Contrast volume [mL]	150.5 ± 82.8	169.47 ± 97.8	0.3965
Radiation dose [Gycm^2^]	800.4 ± 560.3	883.4 ± 863.9	0.5169

* The data are presented as the mean +/− SD.

**Table 3:** Angiographic data of the study population.

	Non-COVID *	COVID (+) *	*p*-Value
Left main disease	15 (11.9)	8 (16.7)	0.9003
1-vessel disease	27 (21.4)	8 (16.7)	0.9003
2-vessel disease	31 (24.6)	10 (20.8)	0.9003
3-vessel disease	46 (36.5)	18 (37.5)	0.9608
Use of thrombectomy	4 (3.2)	3 (6.3)	0.9003
Use of GpIIb/IIIa	6 (4.8)	4 (8.3)	0.9608
No-reflow post PCI	1 (0.8)	1 (2.1)	0.9608
Dissection post PCI	1 (0.8)	0 (0.0)	0.9003
Thrombus on atherosclerotic plaque	9 (7.1)	5 (10.4)	0.9608
Thrombus in non-culprit artery	0 (0.0)	3 (6.3)	0.1905
TIMI 0 pre PCI	9 (12.2)	8 (24.2)	0.4592
TIMI 1 pre PCI	4 (5.4)	1 (3.0)	
TIMI 2 pre PCI	10 (13.5)	8 (24.2)	
TIMI 3 pre PCI	51 (68.9)	16 (48.5)	
TIMI 0 post PCI	1 (1.4)	1 (3.0)	0.3610
TIMI 1 post PCI	0 (0.0)	0 (0.0)	
TIMI 2 post PCI	0 (0.0)	2 (6.1)	
TIMI 3 post PCI	73 (98.7)	30 (90.9)	
Survival rate	118 (93.7)	33 (68.8)	<0.0001

* Data are presented as number and percentage (%).

**Table 4:** Univariate and multivariate logistic regression models for predictors of mortality.

		*p*-Value
Univariate logistic regression *		
Preparation time [min]	1.08 (1.03–1.14)	0.0013
Leaving time (end of procedure to departure) [min]	1.03 (1.01–1.06)	0.0175
Overall cathlab time [min]	1.02 (1.01–1.03)	0.0017
Age	1.07 (1.02–1.12)	0.0077
Prior PCI (0—no. 1—yes)	0.23 (0.05–1.03)	0.0547
COVID (+)—1. (-)—0	6.71 (2.62–17.18)	0.0001
Multivariate logistic regression *		
Age	1.06 (1.01–1.11)	0.0255
COVID (+)—1. (-)—0	5.01 (1.83–13.69)	0.0017

* Data are presented as odds ratio (OR) with 95% confidence interval (95% CI).

## Data Availability

The data presented in this study are available on request from the corresponding author.
